# Modeling the Risk of Team Sport Injuries: A Narrative Review of Different Statistical Approaches

**DOI:** 10.3389/fphys.2019.00829

**Published:** 2019-07-09

**Authors:** Joshua D. Ruddy, Stuart J. Cormack, Rod Whiteley, Morgan D. Williams, Ryan G. Timmins, David A. Opar

**Affiliations:** ^1^School of Behavioural and Health Sciences, Australian Catholic University, Melbourne, VIC, Australia; ^2^Aspetar Orthopaedic and Sports Medicine Hospital, Doha, Qatar; ^3^School of Health, Sport and Professional Practice, Faculty of Life Sciences and Education, University of South Wales, Treforest, United Kingdom

**Keywords:** sport, injury, prevention, prediction, association

## Abstract

Injuries are a common occurrence in team sports and can have significant financial, physical and psychological consequences for athletes and their sporting organizations. As such, an abundance of research has attempted to identify factors associated with the risk of injury, which is important when developing injury prevention and risk mitigation strategies. There are a number of methods that can be used to identify injury risk factors. However, difficulty in understanding the nuances between different statistical approaches can lead to incorrect inferences and decisions being made from data. Accordingly, this narrative review aims to (1) outline commonly implemented methods for determining injury risk, (2) highlight the differences between association and prediction as it relates to injury and (3) describe advances in statistical modeling and the current evidence relating to predicting injuries in sport. Based on the points that are discussed throughout this narrative review, both researchers and practitioners alike need to carefully consider the different types of variables that are examined in relation to injury risk and how the analyses pertaining to these different variables are interpreted. There are a number of other important considerations when modeling the risk of injury, such as the method of data transformation, model validation and performance assessment. With these technical considerations in mind, researchers and practitioners should consider shifting their perspective of injury etiology from one of reductionism to one of complexity. Concurrently, research implementing reductionist approaches should be used to inform and implement complex approaches to identifying injury risk. However, the ability to capture large injury numbers is a current limitation of sports injury research and there has been a call to make data available to researchers, so that analyses and results can be replicated and verified. Collaborative efforts such as this will help prevent incorrect inferences being made from spurious data and will assist in developing interventions that are underpinned by sound scientific rationale. Such efforts will be a step in the right direction of improving the ability to identify injury risk, which in turn will help improve risk mitigation and ultimately the prevention of injuries.

## Introduction

Injuries are a common occurrence in team sports such as Australian football (Australian Football League, 2017), soccer (Ekstrand et al., 2011) and rugby (Fuller et al., 2013). The incidence rates in these sports can impose a significant financial burden on individual athletes and their sporting organizations (Woods et al., 2002; Cumps et al., 2008; Hickey et al., 2014). Additionally, injuries can impact team and individual performances (Verrall et al., 2006; Hagglund et al., 2013; Podlog et al., 2015; Drew et al., 2017), as well as physical and psychological wellbeing (Rozen et al., 2007). Due to these high injury rates and resulting costs, an abundance of research has attempted to identify factors that may increase or decrease athletes’ risk of injury (Orchard, 2001; Hoskins and Pollard, 2003; Arnason et al., 2004; Gabbett, 2005; Opar et al., 2012; Freckleton and Pizzari, 2013), which is important when developing prevention and risk mitigation strategies (van Mechelen et al., 1992; Bahr and Holme, 2003; Arnason et al., 2004). There are a number of methods that can be used to identify factors that are associated with injury risk (Bahr and Holme, 2003; McCall et al., 2017). However, there is a level of confusion that can result from practitioners misinterpreting the different statistics that are often reported (McCall et al., 2017). For example, a lack of understanding in relation to direct and indirect association (discussed in section “Association Versus Prediction” of this narrative review) may result in practitioners concluding that a factor associated with injury risk can be used to predict (and ultimately prevent) injury (McCall et al., 2017; Smoliga and Zavorsky, 2017; Stovitz et al., 2019). In turn, this may lead to incorrect inferences being made from spurious data (Smoliga and Zavorsky, 2017). For this reason, it is important to understand the differences and nuances between association and prediction when interpreting research and making inferences from data (Shmueli, 2010; McCall et al., 2017).

In the context of injuries, association can help us understand why an injury occurs (Altman and Krzywinski, 2015; McCall et al., 2017). Studies exploring association can identify whether a relationship exists between a certain factor and the risk of injury and can provide information about injury risk at a group level (Altman and Krzywinski, 2015; McCall et al., 2017). However, as previously highlighted it is important to understand that associations can occur as a result of indirect, intermediate variables, as well as complete luck (Smoliga and Zavorsky, 2017; Stovitz et al., 2019). Such distinctions are critical when interpreting and making decisions from data (McCall et al., 2017; Smoliga and Zavorsky, 2017). Prediction, in the current context, is the ability to identify injury risk, as a whole, and predict outcomes at an individual level (Shmueli, 2010). A factor that is highly associated with the risk of injury cannot necessarily be used to predict injury at the individual level (Shmueli, 2010; McCall et al., 2017). For example, statistically derived cut points for screening tests, discussed later in this narrative review, may provide information in regards to the risk of injury at a group level (i.e., athletes above or below the cut point), but will most likely perform poorly if used to identify individual athletes that will sustain an injury (Bahr, 2016). This poor performance is likely due to the complex nature of injury etiology. Injuries occur as a result of complex and non-linear interactions between multiple factors (Bittencourt et al., 2016) and it is unlikely that a single, isolated factor is capable of providing enough information to predict injuries at the individual level (Bahr, 2016).

There is also always a level of uncertainty when it comes to injuries. Acute injuries occur following an inciting event and this event may be extrinsic, such as contact with another player, or intrinsic, such as jumping or changing directions (Meeuwisse et al., 2007). Due to the highly unpredictable nature of team sports, the ability to predict the occurrence of an inciting event (e.g., contact with another player) and subsequently an injury, is highly unlikely. As such, the ultimate goal of predictive modeling in sports injury prevention, should not be to predict the occurrence of an injury. Instead, the aim should be to identify injury risk at an individual level and to implement interventions to mitigate the level of risk (Meeuwisse et al., 2007). The ability to mitigate injury risk, however, depends on identifying factors that are associated with injury risk and understanding the methods that can be employed to do so. A better understanding of the different approaches that can be implemented when modeling the risk of sports injuries may improve the ability to identify injury risk. In turn, this may lead to a better understanding as to why they occur and ultimately help improve risk mitigation and injury prevention strategies. Accordingly, the aims of this narrative review are to 1) outline commonly implemented methods for determining injury risk, 2) highlight the differences between association and prediction as it relates to injury and 3) describe advances in statistical modeling and the current evidence relating to predicting injuries in sport.

## Determining Factors That Are Associated With Injury Risk

Methods that are used to determine injury risk factors typically involve classifying athletes as sustaining an injury or remaining injury free, based on the presence or absence of a variable of interest (or injury risk factor). This is referred to as binary classification. There are four possible outcomes in binary classification:

• True positive (TP) = the variable of interest was present and the athlete was injured.
• False positive (FP) = the variable of interest was present but the athlete avoided injury.
• True negative (TN) = the variable of interest was absent and the athlete avoided injury.
• False negative (FN) = the variable of interest was absent but the athlete was injured.

**FIGURE 1:**
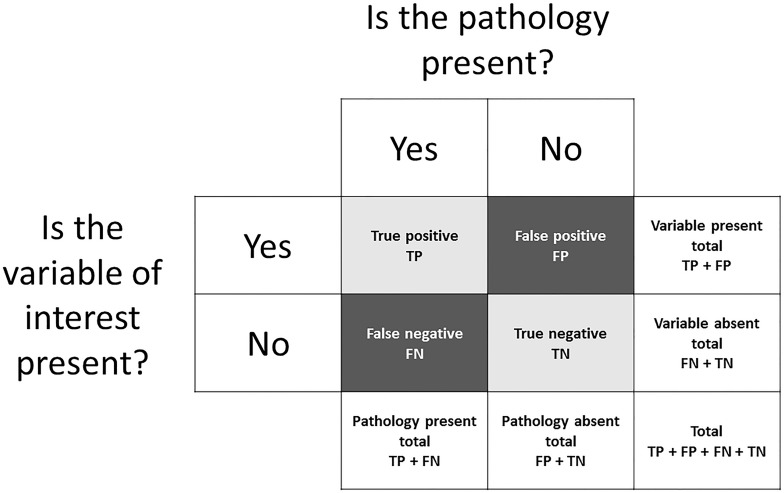
A contingency table which can be used to express the outcomes of binary classification.

**FIGURE 2:**
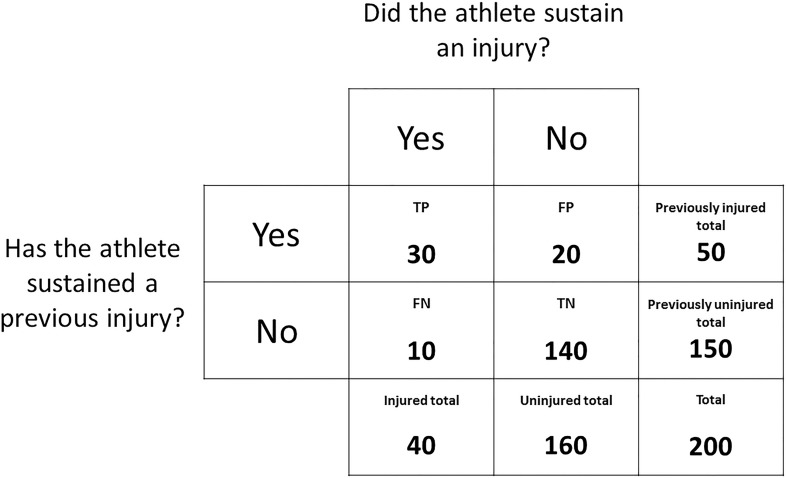
A contingency table expressing the outcomes of a mock dataset. The frequency distribution of athletes that have or have not sustained a previous injury is displayed against the frequency distribution of athletes that did or did not sustain a prospective injury.

These outcomes can be expressed in a contingency table (Figure 1). For the purpose of explaining methodologies that can be used to determine factors associated with injury risk, a mock dataset has been outlined in Figure 2. Given previous injury is commonly associated with the risk of future injury (among a multitude of other factors) (Hägglund et al., 2006), this mock dataset consists of the number of previously injured athletes and the number of prospectively injured athletes. This dataset will be used as an example throughout section “Determining Factors that Are Associated With Injury Risk” of this narrative review and should be referred to alongside Figure 1. The readers should note that the calculations throughout section “Determining Factors that Are Associated With Injury Risk” can be replicated with other dichotomous risk factors.

### Relative Risks and Odds Ratios

Relative risks and odds ratios are commonly used in medical literature to describe the association between a variable of interest and an outcome. Before understanding relative risks and odds ratios, it is important to understand the difference between probability (used to calculate relative risks) and odds (used to calculate odds ratios). Probability is the likelihood of an injury occurring, with zero indicating no chance of an injury occurring and one indicating an injury will certainly occur (Grimes and Schulz, 2008). An advantage of probability is that it can be expressed as a percentage which is easily understood. Using the data in Figure 2, the probability of an injury occurring is calculated as:

$$P\,r\,o\,b\,a\,b\,i\,l\,i\,t\,y=\frac{T\,P+F\,N}{T\,P+F\,P+F\,N+T\,N}$$

$$\frac{30+10}{30+20+10+140}=0.20$$

A probability of 20% suggests that one in five athletes were likely to sustain an injury. Odds, however, is a ratio of the likelihood of an injury occurring compared to the likelihood of an injury not occurring, and is therefore calculated differently to probability (Grimes and Schulz, 2008). Odds can range between zero and infinity and is calculated as:

$$O\,d\,d\,s=\frac{T\,P+F\,N}{F\,P+T\,N}$$

$$\frac{30+10}{20+140}=0.25$$

Odds of 0.25 actually indicates a ratio of 1:4, which means that for every one athlete that sustains an injury, four athletes will remain uninjured. This may be misinterpreted as equaling a probability of 25%. However, a probability of 20% and odds of 0.25 ultimately indicate the same likelihood.

Relative risk, in the current example, is the ratio of the probability of injury occurring in the previously injured group compared to the probability of injury occurring in the previously uninjured group (Schmidt and Kohlmann, 2008). The relative risk is calculated as:

$$R\,e\,l\,a\,t\,i\,v\,e\,r\,i\,s\,k=\frac{T\,P÷\left(T\,P+F\,P\right)}{F\,N÷\left(F\,N+T\,N\right)}$$

$$\frac{30÷\left(30+20\right)}{10÷\left(10+140\right)}=9.0$$

Using the current data (Figure 2), the relative risk is 9. This means that athletes with a previous injury had a 9-fold higher chance of sustaining a future injury than athletes without a previous injury. A relative risk of 1.0 would suggest that the probability of future injury was the same for both athletes with or without a previous injury and that previous injury is not associated with the risk of future injury. A relative risk of 0.5 would indicate that the previously injured athletes had half the chance of sustaining a future injury when compared to the previously uninjured athletes.

In the current example, the odds ratio is the ratio between the odds of injury occurring in the previously injured group compared to odds of injury occurring in the previously uninjured group (Schechtman, 2002). The odds ratio is calculated as:

$$O\,d\,d\,s\,r\,a\,t\,i\,o=\frac{T\,P÷F\,N}{F\,P÷T\,N}$$

$$\frac{30÷10}{20÷140}=21.0$$

Using the data in Figure 2, the odds ratio is 21. This means that the odds of the previously injured athletes sustaining an injury in the current sample were 21 times higher than the odds of the previously uninjured athletes sustaining an injury. Since relative risks and odds ratios primarily consider the frequencies of a risk factor and the rate of injury, respectively, the difference between these two statistics is dependent on the relative frequencies of each of these elements. While these differences may be subtle, they can also be misleading. In the current example, the same data can be presented as a relative risk of 9 or an odds ratio of 21, which may suggest a greater increase in the level of risk. These two metrics, however, are not equivalent and should not be interpreted as such. Compared to relative risks, odds ratios are more sensitive to higher initial incidences of the outcome (Schmidt and Kohlmann, 2008). For example, if the initial injury incidence was 30% as opposed to 20% (e.g., TP = 40, FP = 10, FN = 20 and TN = 130), the odds ratio would be 4 times greater than the relative risk, as opposed to 2 times greater when using the data from Figure 2. Given injury incidences can be variable in prospective cohort studies (Bahr and Holme, 2003), calculating the relative risk of injury (as opposed to the odds ratio) is suggested to be the more appropriate method (Schmidt and Kohlmann, 2008).

### Sensitivity and Specificity

Sensitivity and specificity are measures of the performance of a binary classification test (Akobeng, 2007a). Sensitivity, referred to as the true positive rate, measures the proportion of injured athletes that were correctly classified as being injured, while specificity, referred to as the true negative rate, measures the proportion of uninjured athletes correctly classified as such (Akobeng, 2007a). Using the data in Figure 2, sensitivity is calculated as:

$$S\,e\,n\,s\,i\,t\,i\,v\,i\,t\,y=\frac{T\,P}{T\,P+F\,N}$$

$$\frac{30}{30+10}=0.750$$

Specificity is calculated as:

$$S\,p\,e\,c\,i\,f\,i\,c\,i\,t\,y=\frac{T\,N}{T\,N+F\,P}$$

$$\frac{140}{140+20}=0.875$$

The sensitivity indicates that injury history was able to correctly classify 75% of the prospectively injured athletes, while the specificity indicates that 88% of the uninjured athletes were correctly classified. Sensitivity and specificity are often calculated alongside the relative risk and odds ratio to give an indication of how well a variable classified the injured and uninjured athletes at a group level (Akobeng, 2007a). However, in the context of attempting to predict future injuries, sensitivity and specificity are meaningless, as these metrics can only be calculated retrospectively. In order to calculate the sensitivity and specificity of a test, a practitioner needs to know which athletes were injured and uninjured and the purpose of a test for predicting future injuries is to determine this (Whiteley, 2016). Simply put, if we knew who was going to sustain an injury and who wasn’t, we wouldn’t need to apply the test to find out. Accordingly, sensitivity and specificity (along with relative risks and odds ratios) provide no information regarding the predictive ability of a test (or an injury risk factor).

### Pre-test and Post-test Probabilities

The previously discussed methods can be used to identify the influence a variable has on the risk of injury, but only in one group relative to another. They do not take into account the base rate of injury. Bayes’ theorem can be used to explain the likelihood of an event occurring given the baseline probability of that event occurring as well as the introduction of new evidence (the presence or absence of the variable of interest) (Akobeng, 2007b). Pre-test and post-test probabilities (sometimes referred to as prior and posterior probabilities) are a simple application of Bayes’ theorem and can be used to determine the influence a variable has on the probability of injury relative to the base rate of injury and not another group (Akobeng, 2007b). Using the current example of previous injury as a risk factor and the data from Figure 2, the pre-test probability is calculated as previously outlined (see section “Relative risks and odds ratios”):

$$Pre-testprobability=\frac{T\,P+F\,N}{T\,P+F\,P+F\,N+T\,N}$$

$$\frac{30+10}{30+20+10+140}=0.20$$

The next step requires us to transition from probability to odds. The pre-test odds can be calculated using the previously outlined equation (see section “Relative risks and odds ratios”), or can be calculated using the pre-test probability:

$$Pre-testodds=\frac{Pre-testprobability}{1-pre-testprobability}$$

$$\frac{0.2}{1-0.2}=0.25$$

The post-test odds and subsequently the post-test probability can be calculated using likelihood ratios (Ruddy et al., 2016; Whiteley, 2016). The likelihood ratio indicates the magnitude of the effect that injury history has on the odds of sustaining a future injury (Akobeng, 2007b; Whiteley, 2016). The likelihood ratio for athletes with a previous injury (referred to as the positive likelihood ratio) is calculated as:

$$P\,o\,s\,i\,t\,i\,v\,e\,l\,i\,k\,e\,l\,i\,h\,o\,o\,d\,r\,a\,t\,i\,o=\frac{S\,e\,n\,s\,i\,t\,i\,v\,i\,t\,y}{1-s\,p\,e\,c\,i\,f\,i\,c\,i\,t\,y}$$

$$\frac{0.75}{1-0.875}=6.0$$

The positive likelihood ratio indicates that having a previous injury increased the odds of sustaining a future injury 6-fold (see section “Sensitivity and specificity” for sensitivity and specificity calculations). The negative likelihood ratio can also be calculated for athletes without a history of injury, but this is typically less relevant for practitioners that are interested in the impact a variable has on injury risk. The post-test odds of sustaining a future injury is simply the pre-test odds multiplied by our positive likelihood ratio:

Post-testodds=Pre-testodds×positivelikelihoodratio

0.25×6=1.50

Following this, the post-test odds can be used to transition back to probability and to calculate the post-test probability of sustaining a future injury:

$$Post-testprobability=\frac{Post-testodds}{Post-testodds+1}$$

$$\frac{1.5}{1.5+1}=0.60$$

Before considering injury history, the probability of injury for the 200 athletes was 20%, or a 2 in 10 chance. After taking into account injury history (or the ‘new evidence’), the probability of injury for the previously injured athletes increased to 60%, or a 6 in 10 chance. A concise summary of these steps and the calculations involved can be found in Table 1.

**TABLE 1:** A summary of the steps involved in calculating the post-test probability of an injury occurring given a history of injury.

**Step**	**Statistic**	**Value**	**Calculation**	**Description**
1. Pre-test	Odds (as a decimal)	0.25	$\frac{T\,P+F\,N}{F\,P+T\,N}$	The decimal odds of sustaining a future injury for all athletes, prior to accounting for previous injury. This can also be calculated using the pre-test probability (see section “Pre-test and Post-test Probabilities”).
	Odds (as a ratio)	1:4	As above, calculated as a fraction	The likelihood of a future injury occurring (1) compared to the likelihood of a future injury not occurring (4) for all athletes.
	Probability	20%	$\frac{T\,P+F\,N}{T\,P+F\,P+F\,N+T\,N}\times 100$	The percentage of athletes likely to sustain a future injury (prior to accounting for previous injury).
	Explanation	2 in 10 chance	−	This can simplified to a 1 in 5 chance.
2. Likelihood ratio	Positive likelihood ratio	6	$\frac{S\,e\,n\,s\,i\,t\,i\,v\,i\,t\,y}{1-s\,p\,e\,c\,i\,f\,i\,c\,i\,t\,y}$	The magnitude by which having a previous injury increases the odds of sustaining a future injury. This is calculated using sensitivity and specificity (see section “Sensitivity and Specificity”).
3. Post-test	Odds (as a decimal)	1.5	*Pre*−*testodds*×*positivelikelihoodratio*	The decimal odds of athletes with a previous injury sustaining a future injury.
	Odds (as a ratio)	6:4	As above, calculated as a fraction	The likelihood of a future injury occurring (6) compared to the likelihood of a future injury not occurring (4) for athletes with a previous injury.
	Probability	60%	$\frac{P\,o\,s\,t-t\,e\,s\,t\,o\,d\,d\,s}{P\,o\,s\,t-t\,e\,s\,t\,o\,d\,d\,s+1}\times 100$	The percentage of previously injured athletes likely to sustain a future injury. This is calculated using the post-test odds.
	Explanation	6 in 10 chance	−	This can be simplified to a 3 in 5 chance.

*The derived values have been calculated using the example and mock dataset illustrated in Figure 2. For a detailed outline of the steps involved, see section “Pre-test and Post-test Probabilities.” TP, true positive; TN, true negative; FP, false positive; FN, false negative.*

### Continuous Variables

Up to this point, previous injury has been used as an example to explain methodologies that can be used to determine the association between a factor and the risk of injury. However, in the current example, injury history is a binary categorical variable. This means there are only two possible options: previously injured or previously uninjured. Such data can be easily expressed in a contingency table (Figure 1). Factors associated with an increase or decrease in the risk of injury, however, are often continuous in nature and not binary (Akobeng, 2007c). Continuous variables, such as anthropometric characteristics, running distances or muscular strength, are measured and can result in any value within a feasible range. In order to express these data in a contingency table and implement the previously discussed methods, a cut point must be selected and athletes must be classified as being either above or below the cut point. This cut point may be chosen arbitrarily, or it may depend on the distribution of the data (i.e., above or below the mean). Alternatively, a more pertinent option is to select the cut point which maximizes sensitivity and specificity (Akobeng, 2007c). This can be done using a receiver operating characteristic (ROC) curve.

The ROC curve was first developed during the Second World War and was used to analyze the classification accuracy of radar operators in distinguishing a signal from noise in radar detection (Streiner and Cairney, 2007). When the sensitivity of the radar was increased, true signals were better detected. However, this also increased the amount of noise picked up and the likelihood of this being misinterpreted as a true signal, the consequence of which often meant death (Streiner and Cairney, 2007). The ROC curve was implemented to maximize the amount of true signals detected while minimizing the amount of noise picked up (Streiner and Cairney, 2007).

**FIGURE 3:**
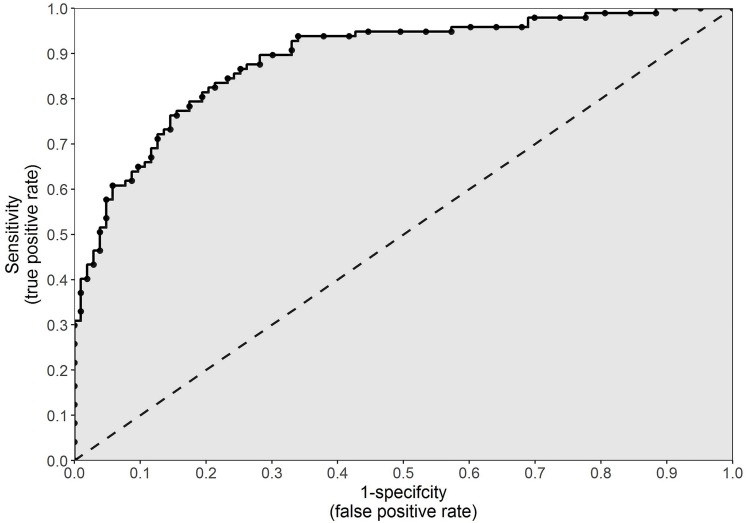
An example of a receiver operating characteristic curve, which can be used to illustrate how well a continuous variable performs as a binary classifier. The true positive rate (sensitivity) is plotted against the false positive rate (1 – specificity) at every conceivable cut point for a continuous variable. The gray shaded area indicates the area under the curve.

More recently, ROC curves have been used in the medical sphere for the evaluation of diagnostic tests (Streiner and Cairney, 2007). In the current context of sports injuries, however, a ROC curve can be used to illustrate how well a continuous variable performs as a binary classifier (i.e., injured or uninjured) (Akobeng, 2007c). A ROC curve can be created by plotting the true positive rate (sensitivity) against the false positive rate (1 – specificity) at every conceivable cut point for a continuous variable (Figure 3). The false positive rate, which is the inverse of specificity, is the proportion of athletes incorrectly classified as prospectively injured. The perfect cut point for a continuous variable would result in 100% sensitivity and 100% specificity (that is, all injured and uninjured athletes correctly classified as such). However, as illustrated in Figure 3, an increase in sensitivity will typically result in an increase in 1 – specificity (or a decrease in specificity) (Akobeng, 2007c). The cut point which results in sensitivity closest to 1 and 1 – specificity closest to 0 (top left-hand corner of the graph illustrated in Figure 3), will be the cut point which maximizes the number of correct classifications while minimizing the number of incorrect classifications (Akobeng, 2007c). This can be determined as the cut point which yields the closest value to 1 given the following formula:

S⁢e⁢n⁢s⁢i⁢t⁢v⁢i⁢t⁢y-(1-s⁢p⁢e⁢c⁢i⁢f⁢i⁢c⁢i⁢t⁢y)

An optimal cut point, however, is highly specific to the spread of the data from which it is derived. While it can provide information about a variable and its application to a specific cohort, in reality, a statistically derived cut point has little clinical relevance. A more important use of a ROC curve is the ability to calculate the area under the ROC curve, commonly referred to as simply area under the curve (AUC). The higher the sensitivity and the lower the 1 – specificity at every point on a ROC curve, the greater the AUC will be. Illustrated in Figure 3 as the gray shaded area, AUC is an overall measure of how well a continuous variable (not a specific cut point for that variable) can distinguish between the prospectively injured and uninjured athletes (Akobeng, 2007c). For example, if we operate under the assumption that greater running distances increase the risk of injury, then we would assume that the injured athletes should have run further than the uninjured athletes. The AUC is equal to the proportion of cases in which this assumption proves to be true (Akobeng, 2007c). An AUC of 0.5 (illustrated as the area below the 45 degree line in Figure 3) indicates classification no better than random chance, whereas 1.0 indicates perfect classification. An AUC of less than 0.5 suggests that the assumption regarding the direction of our continuous variable is incorrect. In the previous example, a value less than 0.5 would indicate that lesser running distances, not greater, increase the risk of future injury. There are no formal guidelines, however, as to interpreting which values indicate good or poor performance.

## Association Versus Prediction

The methodologies discussed up to this point are appropriate for investigating the association between a variable and the risk of injury. It can be assumed that two variables are associated when one variable provides information about the other (Altman and Krzywinski, 2015; Stovitz et al., 2019). Studies investigating association are important due to their ability to identify injury risk factors and provide important information regarding the etiology of injuries (Bahr and Holme, 2003). Research of this nature can help us understand why an injury occurs, either directly or indirectly (McCall et al., 2017). If one variable causes a specific outcome in another variable, then the two variables are associated and direct causation can be inferred (Altman and Krzywinski, 2015; Stovitz et al., 2019). However, two variables can still be associated even in the absence of direct causation (Altman and Krzywinski, 2015; Stovitz et al., 2019). Indirect causation occurs when one variable influences the outcome of another variable via an intermediate variable (Altman and Krzywinski, 2015; Stovitz et al., 2019). Two associated variables may simply have a common cause or even a common consequence, rather than a direct link (Altman and Krzywinski, 2015; Stovitz et al., 2019). For example, we might observe a strong association between going to bed with our shoes still on and waking up with a headache the following morning. However, simply ensuring we remove our shoes before we go to bed won’t prevent a headache the following morning. Both of these events are much more likely mediated by the amount of alcohol consumed before going to bed. From a sports injury perspective (adapted from Stovitz et al., 2019), we might observe an increase in the risk of sustaining an injury with an increase in the number of goals scored from penalties during a soccer match. However, reducing the amount of goals scored from penalties by recruiting a world-class goalkeeper likely won’t reduce the risk of injury. Injury risk, as well as the number of goals scored from penalties, will instead be directly associated with the number of dangerous tackles for which the referee calls a penalty (Stovitz et al., 2019). There is also the possibility that the correlation between two variables is simply the product of luck and not a meaningful relationship. This occurrence is referred to as a type I error. A type I error occurs when an effect is inferred, when no effect exists in reality. A type II error, however, occurs when no effect is inferred, when in reality there is an effect. In regards to type I errors, one study has observed that National Football League teams with animals depicted in their logos were at a reduced risk of concussion compared to teams without animal logos (Smoliga and Zavorsky, 2017). Based on these data, it is suggested (satirically) that teams should consider changing their logos in order to reduce the risk of concussion (Smoliga and Zavorsky, 2017).

Difficulties in understanding the nuances of association versus prediction may result in practitioners concluding that a factor associated with injury risk can be used to predict (and ultimately prevent) injury (McCall et al., 2017). In turn, this may lead to incorrect inferences being made from spurious data. In the context of injuries, association can help us identify individual pieces to the overall puzzle of why injuries occur, but only at a theoretical (i.e., group) level. Prediction, however, is the ability to apply the theoretical framework (or the overall puzzle) at an individual level and make predictions from known values to unknown outcomes (Shmueli, 2010). A practical example of the discrepancies between association and prediction, is hamstring strain injury (HSI) risk in elite Australian footballers. A group of 186 elite Australian footballers, competing in the 2013 AFL season, had their eccentric hamstring strength measured at the start of pre-season via a field testing device (Opar et al., 2015). Athletes below 256 N of eccentric hamstring strength were found to be at an increased risk of HSI throughout the season, relative to the athletes above this cut point (Opar et al., 2015). This cut point produced the highest sensitivity and specificity (0.63 and 0.65 respectively) and resulted in a relative risk of 2.7 (95% confidence interval = 1.3–5.5) (Opar et al., 2015). Additionally, significant interactions between eccentric hamstring strength, age and previous HSI data were observed, with the authors concluding that these factors examined collectively can better assist in assessing an individual’s risk of HSI (Opar et al., 2015). It may be tempting to conclude that 256 N as a cut point for eccentric hamstring strength offers some predictive ability when it comes to HSIs in elite Australian footballers. However, this cut point was determined retrospectively from the data it was applied to and as a result, is closely fit to the data from which it was derived. Therefore, the cut point may appear to display some level of predictive capacity within that particular cohort. In reality, however, inferences regarding the predictive ability of the cut point cannot be made without applying it to another cohort from which it was not a derivative of (Bahr, 2016). These methodologies (i.e., reductionist methodologies) are useful in establishing a link between certain factors and the risk of injury, but they cannot be used to predict injury (Bahr, 2016). A more recent study replicated the same data collection methods two years following the original investigation (2015 AFL season) (Ruddy et al., 2017). This study used the dataset from the 2013 season, to build a model with the aim of predicting HSI outcomes during the 2015 season (Ruddy et al., 2017). To investigate the predictive ability of age, previous HSI and eccentric hamstring strength data, this study employed a machine learning approach (i.e., a complex approach), which will be discussed later in this narrative review. It was concluded, however, that despite these risk factors showing an association with the risk of HSI, these data could not be used to predict the occurrence of HSI with any consistency (Ruddy et al., 2017). Other research has highlighted the need for data to be shared and for studies to be replicated, so that results can be verified and casual effects, rather than coincidental effects, can be established (Nuzzo, 2015; Smoliga and Zavorsky, 2017; van Dyk et al., 2017).

### Reductionist Versus Complex Approaches

It has long been suggested that a univariable approach (that is, investigating a single variable’s impact on injury risk) may be too simplistic and that in order to better understand the etiology of injuries, the collective contribution of multiple factors to injury risk must be examined (a multivariable approach) (Bahr and Holme, 2003; Quatman et al., 2009; Mendiguchia et al., 2012; Nielsen et al., 2016). A similar study to those aforementioned, investigating HSI risk in elite soccer players, employed a multivariable approach to identify HSI risk (Timmins et al., 2016). Biceps femoris fascicle length, eccentric hamstring strength, injury history and age were all examined in concert to determine the collective impact of these variables on HSI risk (Timmins et al., 2016). Despite implementing a multivariable approach, this study found that these variables only accounted for approximately 30% of the risk associated with HSI (Nagelkerke R^2^ coefficient = 0.31) (Timmins et al., 2016). It is becoming widely accepted that injuries occur as a result of complex and non-linear interactions amongst multiple variables and that conventional approaches, even multivariable ones, are unlikely to capture the dynamic and multiplex nature of injuries (Quatman et al., 2009; Bittencourt et al., 2016; Ruddy et al., 2016). Therefore, it has been proposed that researchers and practitioners alike need to shift their perspective of injury etiology from one of reductionism to one of complexity (Quatman et al., 2009; Mendiguchia et al., 2012; Bittencourt et al., 2016).

The previously discussed statistical approaches are reductionist in nature. Reductionism assumes that all the parts of a system (in this case, injury etiology) can be broken down and examined individually and then summed together to represent the system as a whole (Quatman et al., 2009). Quatman et al. (2009) describe it as examining the individual pieces of a bike and assuming you understand how all the pieces fit together to operate as a whole system. If you have never seen a bike before, your interpretation of how all the pieces fit together to create the bike and how the bike then operates may be wildly inaccurate (Quatman et al., 2009). Even when implementing a multivariable approach, conventional methods are still limited by the assumption that a system is equal to the sum of its parts (Mendiguchia et al., 2012). A reductionist approach is useful as it allows us to identify and focus on the individual parts of a system (in this case, injury risk factors) even if it fails to capture the complex, non-linear interactions which occur amongst the individual parts to form the whole system (Bittencourt et al., 2016). Studies employing reductionist approaches can be used, however, to inform and implement complex approaches in future research (Bittencourt et al., 2016). Simply put, the relevance of a variable should be determined via reductionist (or conventional) approaches, prior to being used to predict specific outcomes.

**FIGURE 4:**
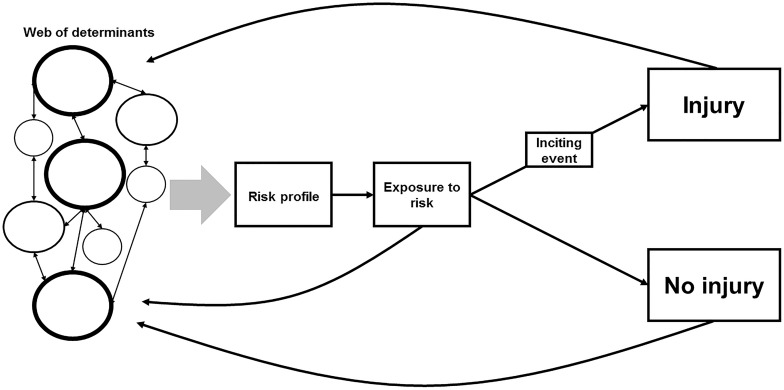
A complex systems approach for modeling the risk of injury, adapted from Bittencourt et al. (2016). The web of determinants represents the individual risk factors as a collective, with the size of the circle indicating that variable’s level of influence. These variables interact with each other to differing degrees to result in a risk profile. An athlete is then exposed to the risk of injury during training/competition. During an athlete’s exposure to the risk of injury, an inciting event may occur and this can result in an injury. The other outcome is no injury. How much an athlete trains or competes and what they do during these sessions (i.e., their level of exposure) will feed back into the web and revise their subsequent and resulting risk profile. The outcome (injury or no injury), will also influence their future risk profiles.

Bittencourt et al. (2016) propose a complex, albeit theoretical, model for injury etiology. This model has been adapted and graphically represented in Figure 4. At the beginning of the model, we have what has been coined as the web of determinants (Philippe and Mansi, 1998; Bittencourt et al., 2016). The web is made up of individual factors, some of which contribute to the risk of injury to greater extents (as indicated by the size of the circles and their borders). All these factors interact with other factors to differing degrees to form the risk profile. This risk profile is an individual athlete’s level of predisposed injury risk. An athlete must be exposed to the risk of injury, however, and this exposure occurs during training or competition. If an athlete is not exposed to the risk of injury, the likelihood of an injury occurring is near zero. During an athlete’s exposure, an inciting event may occur and this can result in an injury. The other outcome is no injury. How much an athlete trains or competes and what they do during these sessions (i.e., their level of exposure) will feed back into the web and revise their subsequent and resulting risk profile. The outcome (injury or no injury) will also influence their future risk profiles. This model, however, is theoretical and a complex approach can be difficult to implement in a practical setting (Meeuwisse et al., 2007; Bittencourt et al., 2016). It has been suggested, however, that machine learning may be an appropriate option when applying this complex model to the real world (Quatman et al., 2009; Bittencourt et al., 2016).

### Machine Learning

**FIGURE 5:**
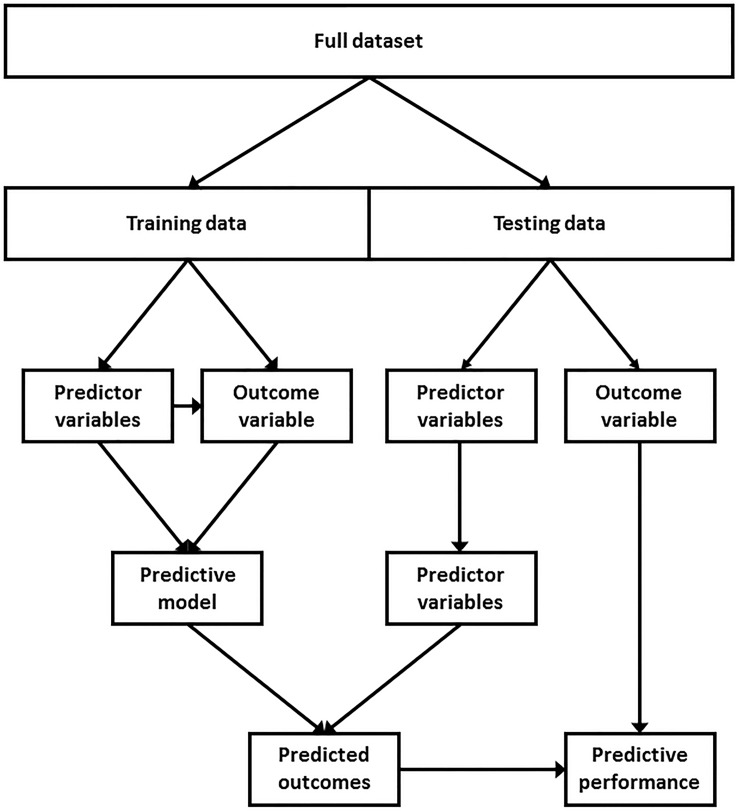
A typical supervised learning modeling approach. A dataset with a known outcome variable (i.e., injured or uninjured), referred to as training data, is used to identify patterns and predict the withheld outcome variable of an independent dataset, referred to as testing data. The performance of the model can then be assessed by comparing the predicted outcomes against the withheld outcome variable of the testing data.

Machine learning is a field of computer science which involves building algorithms to learn from data and make predictions without being programmed what to look for or where to look for it. Machine learning techniques can be either supervised or unsupervised. Unsupervised learning is the process by which predictions are made on a dataset with no corresponding outcome variable (Kotsiantis, 2007). However, in prospective injury studies, the outcome variable (injury or no injury) is typically known. Therefore, supervised learning is more relevant to injury research. Supervised learning is the process by which a dataset with a known outcome variable, referred to as training data, is used to identify patterns and predict the same, yet withheld, outcome variable of an independent dataset, referred to as testing data (Figure 5; Han and Kamber, 2006). The training data is used to build the model whereas the testing data is used to measure the predictive performance of the model on unseen data, or data that was not used to build the model. A greater amount of training data will better allow the machine learning algorithm to learn from the data and identify complex and non-linear patterns, if such patterns exist. A dataset, however, can be split into training and testing data a number of different ways. For example, if the dataset consists of multiple seasons, all prior seasons can be used as training data to predict the outcomes of the most current season. Alternatively, the data can be randomly sampled and a certain proportion can be allocated as training data, with the remaining data used as testing data. There is no consensus as to how much data should be used as training data. However, one study suggests that using anywhere between 40 and 80% as training data will likely result in optimal predictive performance (Dobbin and Simon, 2011).

There are a number of different types of algorithms that can be used to build predictive models (Kotsiantis, 2007). Each algorithm has different underlying mathematical functions, as well as a unique set of parameters that can be controlled to determine how the algorithm interacts with and learns from data (Kotsiantis, 2007). Some algorithms are more robust and are less influenced by small nuances in data, whereas others are more complex and sensitive. Different types of algorithms are suited to different types of data, but it is typically good practice to implement a number of different algorithms and compare performances (Kotsiantis, 2007). It is beyond the scope of this narrative review to explain the different mathematical functions of the algorithms. However, readers are directed to additional sources for further information (Kotsiantis, 2007). Below are a list of commonly implemented algorithms for binary (i.e., yes or no) prediction:

• Naïve Bayes
• Logistic regression
• Decision tree
• Random forest
• K-nearest neighbors
• Generalized estimating equation
• Support vector machine
• Neural network

When using different algorithms to build a predictive model, the goal is to select a combination of parameters that optimize an algorithm’s ability to perform on the testing data (Bergstra and Bengio, 2012). Finding the optimal parameter combination will typically involve applying all potential combinations to the data and comparing the performance of each (Bergstra and Bengio, 2012). One potential issue that should be considered when selecting the optimal parameter combination is overfitting. Overfitting occurs when the combination of parameters selected are fit too closely to the training data from which they are ultimately derived (Kotsiantis, 2007). This reduces the generalisability of a model and its ability to perform on unseen testing data (Kotsiantis, 2007). One solution to over-fitting is cross-validation (Bergstra and Bengio, 2012). A common type of cross-validation, known as *k*-fold cross-validation, splits the training data into *k* equal subsets. One of the subsets is withheld and the remaining subsets are used to search for the optimal parameter combination. The selected parameter combination is then validated using the withheld subset. This process is repeated *k*-fold, with each subset being withheld once as the validation subset. The parameter combination that performs the best, on average, across each fold is then selected for the final model.

There are a number of other important considerations when implementing a machine learning approach. When using a variety of continuous variables as predictors, machine learning algorithms can be sensitive to the vastly different scales and magnitudes of the different variables (Han and Kamber, 2006). For example, 2 m could be considered large for an athlete in terms of stature, but nothing at all in terms of running distance. Therefore, it is important to consider transforming all continuous data prior to modeling them. The simplest method of data transformation is normalization (Han and Kamber, 2006). Normalization is the process of scaling values so that they lie within a specific range, typically one to zero. Data are normalized using the following equation:

$$x^{1}=\frac{x-m\,i\,n}{m\,a\,x-m\,i\,n}$$

Where *x* equals the original value, *min* equals the minimum value in the sample, *max* equals the maximum value in the sample and *x*^1^ equals the normalized value.

Normalization, however, can result in a loss of information, particularly when it comes to outliers (Han and Kamber, 2006). Therefore, particularly in machine learning, standardization is a more appropriate option. Standardization involves transforming data so that the mean is equal to zero and the standard deviation is equal to one. A standard score is more commonly known as a z-score. Standard scores are calculated using the following equation:

$$z^{1}=\frac{z-\bar{z}}{s}$$

Where *z* equals the original value, $\bar{z}$ equals the sample mean, *s* equals the sample standard deviation and *z*^1^ equals the standardized value.

Another machine learning challenge is class imbalance (Japkowicz and Stephen, 2002). Class imbalance is highly specific to predictive modeling of injuries. Although injury rates in sports such as Australian football, soccer and rugby may be considered high (Orchard et al., 2013), the number of uninjured athletes will likely always outweigh the number of injured athletes. In the case of a class imbalance, a predictive model can achieve high accuracy by always predicting the over-represented class (in this case, uninjured) (Chawla et al., 2002). However, the high accuracy would only be reflective of the underlying class distribution. A common method of dealing with imbalanced classes is resampling. Resampling can involve both over-sampling and under-sampling. Under-sampling is the process of randomly removing a proportion of the over-represented class, whereas over-sampling is the process of randomly copying and adding a proportion of the under-represented class. An alternative solution is synthetic minority over-sampling technique (SMOTE), which is a combination of both over- and under-sampling (Chawla et al., 2002). Rather than copy already existing data points from the under-represented class, SMOTE synthetically creates new data points which have similar features to that class (Chawla et al., 2002). Implementing techniques such as cross-validation and SMOTE may improve the predictive performance of a model. However, the method of evaluating predictive performance (see section “Evaluating Predictive Performance”) also needs to be considered carefully.

### Evaluating Predictive Performance

When predicting whether an athlete will sustain an injury or remain uninjured, the predicted outcomes versus the actual outcomes can be expressed in a contingency table, similar to Figure 2. Rather than tabulating the number of prospective injuries versus the presence/absence of the variable of interest, the number of predicted injuries can be referenced against the number of actual injuries. In the case of prediction:

• True positive (TP) = the athlete was predicted as injured and was injured.
• False positive (FP) = the athlete was predicted as injured but avoided injury.
• False negative (FN) = the athlete was predicted as uninjured but was injured.
• True negative (TN) = the athlete was predicted as uninjured and avoided injury.

**FIGURE 6:**
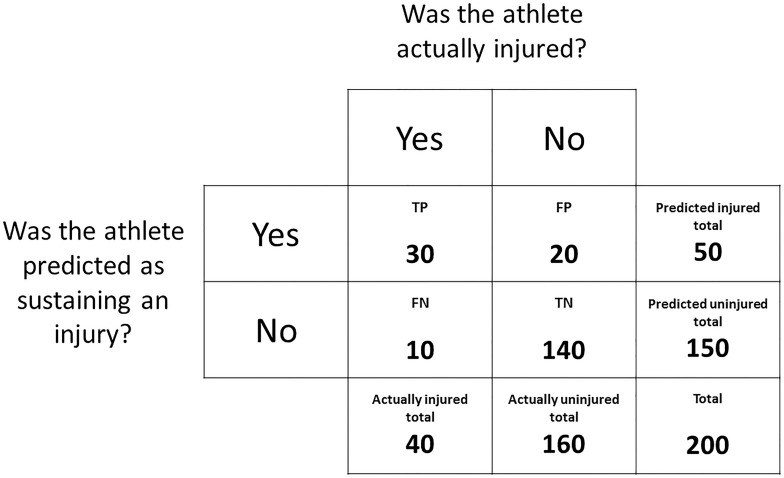
A contingency table expressing the outcomes of a mock dataset. The frequency distribution of athletes predicted as sustaining an injury and athletes predicted as remaining uninjured is displayed against the frequency distribution of athletes that were actually injured and uninjured.

Accuracy is the simplest metric that can be used to evaluate the performance of a predictive model (Han and Kamber, 2006). Accuracy is simply the percentage of correct predictions and, using the data from Figure 6, is calculated as:

$$A\,c\,c\,u\,r\,a\,c\,y=\frac{T\,P+T\,N}{T\,P+F\,P+F\,N+T\,N}$$

$$\frac{30+140}{30+20+10+140}=0.85$$

Accuracy, however, is a poor indicator of performance when the class distribution is not equal, as a predictive model can achieve high accuracy by always predicting the over-represented class (Chawla et al., 2002). Cohen’s kappa coefficient is an alternative to accuracy that accounts for the base rate of expected accuracy due to random chance. The first step when calculating Cohen’s kappa coefficient is to calculate accuracy as above. The next step, using the data from Figure 6, is to calculate the probability of a true positive occurring by chance (*P*^1^):

$$P^{1}=\frac{T\,P+F\,P}{T\,P+F\,P+F\,N+T\,N}\times \frac{T\,P+F\,N}{T\,P+F\,P+F\,N+T\,N}$$

$$\frac{30+20}{30+20+10+140}\times \frac{30+10}{30+20+10+140}=0.05$$

The next step is to calculate the probability of a true negative occurring by chance (*P*^2^):

$$P^{2}=\frac{F\,N+T\,N}{T\,P+F\,P+F\,N+T\,N}\times \frac{F\,P+T\,N}{T\,P+F\,P+F\,N+T\,N}$$

$$\frac{10+140}{30+20+10+140}\times \frac{20+140}{30+20+10+140}=0.60$$

The overall probability of a correct classification occurring by chance (*P*^3^) is then calculated as:

$$P^{3}=P^{1}+P^{2}$$

0.05+0.60=0.65

Cohen’s kappa coefficient is then calculated as:

$$C\,o\,h\,e\,n^{\prime }\,s\,k\,a\,p\,p\,a\,c\,o\,e\,f\,f\,i\,c\,i\,e\,n\,t=\frac{A\,c\,c\,u\,r\,a\,c\,y-P^{3}}{1-P^{3}}$$

$$\frac{0.85-0.65}{1-0.65}=0.57$$

Both accuracy and kappa, however, are calculated using only the number of correct and incorrect predictions and do not account for the predicted probability of injury. An athlete will be predicted as injured if the model returns a probability of injury greater than 50%. If Athlete A has a 49% probability of injury and Athlete B has 1% probability of injury, both these athletes are more likely to remain uninjured and will be predicted as such. Accuracy and kappa do not account for the fact that Athlete A, despite being predicted as uninjured, was still 48% more likely to sustain an injury than Athlete B. An alternative method that accounts for the magnitude of the probability rather than just the binary prediction is AUC. As outlined previously (see section “Continuous Variables”), a ROC curve can be constructed by plotting the true positive rate against the false positive rate at every conceivable cut point for a continuous variable. In this case, however, the curve can be constructed by plotting the true and false positive rates at every conceivable cut point for the estimated injury probabilities (0% to 100%). The AUC is equal to the proportion of cases where a prospectively injured athlete had a higher estimated probability of injury than an uninjured athlete (Akobeng, 2007c).

## Predictive Modeling and Its Limitations in Sports Injury Research

Machine learning has been used to predict outcomes in a variety of fields for a number of years (Falk et al., 1998; Niska et al., 2004; Farmer et al., 2012). Its use in sports related research, however, is becoming increasingly popular. Machine learning has been used to predict the outcomes of matches in baseball, basketball and soccer (Joseph et al., 2006; Haghighat et al., 2013; Zimmermann et al., 2013; Soto Valero, 2016), although the application of these techniques in modeling the risk of injuries in sport is limited. However, a growing number of studies have attempted to predict injuries using previously established risk factors (Ruddy et al., 2017; Carey et al., 2018). One study, which was previously mentioned, attempted to predict HSI in elite Australian footballers (Ruddy et al., 2017). Age, previous HSI and eccentric hamstring strength data, collected across two AFL seasons, were modeled using machine learning methods in an effort to identify risk and predict HSI (Ruddy et al., 2017). This study also collected additional data, which hasn’t been strongly associated with HSI risk, to investigate whether the inclusion of these variables would improve the predictive performance of the models. When predicting HSIs that occurred within the same season, a median AUC of 0.58 was observed (Ruddy et al., 2017). When data from the 2013 AFL season was used to predict HSIs that occurred during the 2015 AFL season, the median AUC was 0.52 (Ruddy et al., 2017). It was also noted that the inclusion of the additional variables did not improve the performance of the models. The authors also implemented SMOTE in an effort to combat the imbalanced classes. No improvements in performance were observed across all models built using SMOTE-sampled data (Ruddy et al., 2017). This study concluded that eccentric hamstring strength, age and previous HSI data cannot be used to identify Australian footballers at an increased risk of HSI with any consistency (Ruddy et al., 2017).

It is suggested that the lack of predictive performance may be for a number of reasons. Firstly, data were only collected at the beginning of pre-season and it is unknown whether more frequent measures of the variables included in the models would have improved performance. The methods implemented in this study (Ruddy et al., 2017) assume that each athletes’ level of eccentric hamstring strength remained constant throughout the season (or up to the point of HSI). However, it has been suggested that changes in strength are more likely to influence the risk of injury than strength measured at one time point alone (Meeuwisse et al., 2007). Accordingly, it has also been suggested that researchers need to consider the risk factors they are investigating and measure data during a time period that is aetiologically relevant (i.e., looking backward from an injury rather than forward from the beginning of a season) (Meeuwisse et al., 2007). In reality, this approach is difficult to implement, particularly in prospective cohort studies. However, considering the different time courses over which risk factor data are measured is an important consideration when modeling the risk of injury. Additionally, a number of factors that have been associated with the risk of HSI were not included in the models and this is likely to have had a significant impact on the ability to predict HSI occurrence (Ruddy et al., 2017). It was suggested that the large variability in AUC seen with each iteration of the models highlights the fragility of the dataset used, with small changes to the randomly sampled training and testing data vastly influencing the performance (Ruddy et al., 2017). As discussed previously, a larger amount of training data will improve the ability of the algorithms to identify patterns and make more meaningful predictions. It is difficult to estimate the amount of data required to achieve a more precise model. One study suggests that up to 10 events (i.e., injuries) per variable are needed to observe any meaningful predictive performance (Peduzzi et al., 1996). However, the ability to capture large injury numbers is a current limitation of sports medicine research (van Dyk et al., 2017). To overcome this, there has been a call to make datasets from sports injury studies available to other researchers, so that analyses and results can be replicated and verified (Smoliga and Zavorsky, 2017; van Dyk et al., 2017). Collaborative efforts such as this will help prevent incorrect inferences being made from spurious data and will assist in developing interventions that are underpinned by sound scientific rationale (Smoliga and Zavorsky, 2017; van Dyk et al., 2017). Given the limitations of smaller datasets, complex approaches are still likely to provide more informative results than reductionist approaches (Bittencourt et al., 2016). However, results pertaining to analyses of smaller datasets should be interpreted cautiously.

Similar research has also investigated whether training load data could be used to predict non-contact injuries in a single team of elite Australian footballers (Carey et al., 2018). A number of variables were collected on a daily basis across three AFL seasons. These variables included total running distance (m), moderate-speed running distance (m between 18 and 24 km/h), high-speed running distance (m above 24 km/h), PlayerLoad (an accelerometer metric measured in arbitrary units) and ratings of perceived exertion (Carey et al., 2018). Similar machine learning techniques and algorithms were used to build predictive models and a mean AUC of 0.65 was observed (Carey et al., 2018). When the models were used to predict hamstring related injuries only, the mean AUC was 0.72 (Carey et al., 2018). However, the mechanisms of these injuries were unknown and not necessarily acute HSIs and this is likely to have influenced the results. The slight improvements in predictive performance, albeit using different data, suggest that daily observations, as opposed to observations from a single time point, may provide greater insight in regards to the etiology of injuries. However, the authors of this study also conclude that more variables and more data are needed to see any meaningful improvements in predictive capacity (Carey et al., 2018). Further research has also investigated the relationship between training load data and the risk of injury in elite Australian footballers (Colby et al., 2017). This study, however, also included additional variables such as subjective ratings of wellness, history of lower limb pain and years of playing experience (Colby et al., 2017). Improvements in performance were observed with the inclusion of the additional variables, however the estimated injury probabilities were derived using *k*-fold cross-validation, as opposed to a true testing/training split as outlined in Figure 5. The results of this study do, however, suggest that the inclusion of additional variables (given their relevance) is an important step in the direction of developing a more holistic understanding of injury etiology.

When implementing complex approaches to model the risk of injury, the primary considerations for researchers (as well as practitioners contributing to research) should be what data to collect and when to collect them. It has been suggested that in the medical sphere, researchers often use the data available to them to shape research questions or areas of exploration (Harrell, 2015). Ideally, research questions and areas of exploration should be developed and used to inform data collection practices and methodologies (Harrell, 2015). Despite this sentiment, the biggest limitation in implementing complex approaches when modeling the risk of injury remains the amount of data that is required for these methodologies to make meaningful inferences (Carey et al., 2018). As previously discussed, complex approaches are still likely to provide more informative results than reductionist approaches, even in light of the limitations of small data (Bittencourt et al., 2016). However, results should always be interpreted cautiously. To overcome the limitations of small data, researcher and practitioners need to consider sharing data and engaging in collaborative efforts to replicate and validate sports injury research (Smoliga and Zavorsky, 2017; van Dyk et al., 2017). In spite of this, when modeling the risk of injury through the application of machine learning, researchers should carefully consider the technical aspects of any models implemented, such as the method of data transformation, model validation, performance assessment and the impact that class imbalances may have on estimated injury probabilities (discussed in sections “Machine Learning” and “Evaluating Predictive Performance” of this narrative review).

## Practical Recommendations and Conclusion

This narrative review aims to serve as a guide to help the reader understand and implement commonly used methods when modeling the risk of injury in team sports. There are a number of methods that can be used to determine factors that are associated with injury risk (McCall et al., 2017). However, it is important to understand the distinction between association and prediction when reading and interpreting the literature (McCall et al., 2017). Studies investigating association are important due to their ability to identify factors that impact the risk of injury (Bahr and Holme, 2003). Studies implementing reductionist approaches should be used to inform and implement complex approaches in future research (Bittencourt et al., 2016). However, caution should be taken when developing complex approaches based on inferences made from studies investigating association. As outlined in Section “Reductionist Versus Complex Approaches” of this narrative review, the contribution of certain variables to the etiology of injury (as determined from reductionist approaches) may be drastically influenced by the multifaceted, non-linear interactions that a complex approach introduces. The ability to recognize these interactions, however, is the purpose and the advantage of implementing a complex approach. A complex approach will consider how all the pieces fit together to form the overall puzzle. This puzzle can then be applied and used to identify injury risk, as a whole, and predict outcomes at an individual level through the application of methods such as machine learning (Bittencourt et al., 2016). Although current research has demonstrated a limited ability to identify risk and predict injuries at the individual level (Ruddy et al., 2017; Carey et al., 2018), the application of machine learning in sports injury research is still in its infancy. However, there are a number of important considerations, which have been discussed throughout this narrative review, when implementing these approaches in future research:

• The variables that are examined; research implementing reductionist approaches to identifying injury risk factors should be used to inform the inclusion/exclusion of relevant variables when implementing complex approaches to identifying injury risk.
• The types of variables; analyses pertaining to categorical (e.g., binary) variables and continuous variables should be interpreted appropriately.
• The amount of data; a larger amount of observations (i.e., time points) and events (i.e., injuries) will improve the ability to identify patterns (should any patterns exist) and make more meaningful predictions.
• Modeling considerations; the method of data transformation and model validation, as well as the impact that class imbalances may have on a model, should be considered carefully.
• The performance metric used; the performance metric (whether it be predictive or associative) should be considered and interpreted appropriately.
• Data replication and sharing; researchers and practitioners should consider making datasets available to other researchers, so that analyses and results can be replicated and verified.

With these considerations in mind, implementing complex approaches and improving our ability to identify risk and predict injuries may lead to a better understanding as to why they happen (Quatman et al., 2009; Bittencourt et al., 2016) and this in turn can help improve risk mitigation and ultimately the prevention of injuries (Quatman et al., 2009; Bittencourt et al., 2016).

## Author Contributions

JR: conceptual outline, writing and editing of the manuscript. DO, SC, RW, RT, and MW: writing and editing of the manuscript.

## Conflict of Interest Statement

The authors declare that the research was conducted in the absence of any commercial or financial relationships that could be construed as a potential conflict of interest.
